# Appropriateness of specialized care referrals for LBP: a cross-sectional analysis

**DOI:** 10.3389/fmed.2023.1292481

**Published:** 2024-01-05

**Authors:** Janny Mathieu, Marie-Ève Robert, Claude-Édouard Châtillon, Martin Descarreaux, Andrée-Anne Marchand

**Affiliations:** ^1^Department of Anatomy, Université du Québec à Trois-Rivières, Trois-Rivières, QC, Canada; ^2^Faculty of Medicine, Université de Montréal, Montreal, QC, Canada; ^3^Centre intégré universitaire de Santé et de Services Sociaux de la Mauricie-et-du-Centre-du-Québec, Trois-Rivières, QC, Canada; ^4^Division of Neurosurgery, Faculty of Medicine, Université de Montréal, Montreal, QC, Canada; ^5^Department of Human Kinetics, Université du Québec à Trois-Rivières, Trois-Rivières, QC, Canada; ^6^Department of Chiropractic, Université du Québec à Trois-Rivières, Trois-Rivières, QC, Canada

**Keywords:** low back pain, referral appropriateness, specialized services, wait time, cross-sectional study

## Abstract

**Background:**

Low back pain (LBP) accounts for a significant proportion of primary care visits. Despite the development of evidence-based guidelines, studies point to the inefficient use of healthcare resources, resulting in over 60.0% of patients with LBP being referred to spine surgeons without any surgical indication. Centralized waiting lists (CWLs) have been implemented to improve access to specialized care by managing asymmetry between supply and demands. To date, no study has provided data on patients’ clinical profiles and referral patterns to medical specialists for LBP in the context of a publicly funded healthcare system operating a prioritization model. The objective of this study was to evaluate the appropriateness of specialized care referrals for LBP after the implementation of a CWL.

**Methods:**

A retrospective cross-sectional analysis of 500 randomly selected electronic health records of patients who attended the outpatient neurosurgery clinic of the administrative Mauricie-et-Centre-du-Québec region was performed. Inclusion criteria were neurosurgery consultation referrals for adults ≥18 years suffering from a primary complaint of LBP, and performed between September 1st, 2018, and September 1st, 2021. Data relevant for drawing a comprehensive portrait of patients referred to the neurosurgery service and for judging referrals appropriateness were manually extracted.

**Results:**

Of the 500 cases analyzed, only 112 (22.4%) were surgical candidates, while 221 (44.2%) were discharge from the neurosurgery service upon initial assessment. Key information was inconsistently documented in medical files, thus preventing the establishment of a comprehensive portrait of patients referred to the neurosurgery service for LBP. Nevertheless, over 80.0% of referrals made during the study period were deemed inappropriate. Inappropriate referrals were characterized by higher proportion of patients symptomatically improved, presenting a back-dominant chief complaint, exhibiting no objective neurological symptoms, and diagnosed with non-specific LBP.

**Conclusion:**

This study reveals a significant proportion of inappropriate referrals to specialized care for LBP. Further research is needed to better understand the factors that prompt referrals to medical specialists for LBP, and the criteria considered by neurosurgeons when selecting the appropriate management strategy. Recent studies suggest that triaging approaches led by musculoskeletal experts may improve referral appropriateness to specialized care.

## 1 Introduction

Canada’s publicly funded health care system is organized around a 3-level of care delivery model, providing access to a broad range of health services (1). Primary health care serves a dual function, being the first point of contact with health care services, while ensuring continuity of care and coordination with secondary and tertiary care providers when specialized services are needed (1). Access to specialized care poses a challenge, as it relies on highly qualified personnel and equipment that may not be readily available, particularly in remote areas, and in underserved communities (2).

In 2016, Quebec’s Ministry of Health and Social Services (MSSS) launched the *Prioritized Access to Specialized Services* (PASS) program (3), characterized by a set of strategies designed to improve access to specialized care by managing asymmetry between supply and demands (4). Among those strategies, centralized waiting lists (CWLs) have been implemented and consolidate multiple service providers’ and organizations’ waiting lists into a single waiting list for a given specialized service. A CWL operates under a prioritization model, where patients are placed into a queue, through a central intake point, and assigned to medical providers according to their level of need (4). The Service Request Management Center (*Centre de répartition des demandes de services-*CRDS) falls into CWLs’ definition, managing all new referrals sent from primary health care providers to specialized services. The province of Quebec counts 15 CRDSs, distributed across its territory (3). Although promising, the latest MSSS report for the year 2021–2022 indicated that 33.0% of specialist consultations, after referral from a family physician, were not carried out within the expected delay (5). As the public health care system struggles with limited health care resources, this reinforces the importance of the quality of referrals (i.e., referring the right patient, at the right time, to the right service, with the right information) (6) in enabling patients to get a timely access to medical specialists.

As the leading cause of years lived with disability worldwide (7), low back pain (LBP) accounts for a significant proportion of outpatient physician visits (8), and for a significant share of Canada’s health care spending (9). Although several evidence-based clinical guidelines have been published over the years to assist primary care providers with the prevention, evaluation and management of LBP, preventing the use of practices that are harmful or wasteful, while ensuring equitable access to effective and affordable health care for patients with LBP remains a national challenge (10). In most healthcare systems from developed countries, general practitioners are the primary contacts responsible for referring patients with MSK conditions to secondary and tertiary care services, and sometimes to other healthcare professionals (11). However, evidence suggests that usual care for patients with LBP often does not match care endorsed in evidence-based guidelines, leading to overuse of imaging and opioids prescriptions at the expense of self-management strategies and patient education (12–14). Several studies (15–19) also suggest an overreliance on the expertise of medical specialists, indicating that between 62.0 to 85.0% of patients referred to spine surgeons for LBP are not surgical candidates, thus delaying access for patients in need of surgical consultation.

In Mauricie-et-Centre-du-Québec, a CRDS has been implemented in 2018 to manage demands for specialized services within the area served by the Centre intégré universitaire de soins de santé et de services sociaux de la Mauricie-et-du-Centre-du Québec (CIUSSS-MCQ). This CRDS is the only CWL within the province of Quebec to manage all referrals from primary care to specialized services, irrespective of referrals source. The CIUSSS-MCQ’s neurosurgery department also distinguishes itself by managing all surgical consultations for LBP, except for traumas, whereas patients with LBP are distributed between specialists in other institutions. Such particularities offer a unique opportunity to capture relevant information regarding patients referred to specialized care services for LBP. To date, no study has yielded objective data that provide a clear understanding of patients’ clinical profiles and referral patterns to specialized care for LBP following the implementation of a CWL. These data could prove to be an invaluable asset in the development of strategies aimed at improving patients’ care trajectories, while promoting optimal use of health care resources and timely access to medical specialists.

Therefore, our objective was to determine the appropriateness of referrals made to the CIUSSS-MCQ’s neurosurgery service for LBP. This objective was broken down into 3 specific objectives: [1] quantify and describe patients referred for an initial consultation to the neurosurgery department for a primary complaint of LBP; [2] determine the proportion of referrals made to the neurosurgery department for a primary complaint of LBP that is deemed inappropriate; and [3] identify the characteristics that differentiate inappropriate from appropriate referrals.

## 2 Methods

The reporting of this study followed the REporting of studies Conducted using Observational Routinely collected Data (RECORD) checklist (20).

### 2.1 Ethics approval

The Centre intégré universitaire de santé et de services sociaux de la Mauricie-et-du-Centre-du-Québec (CER-2022-600-838) and the Université du Québec à Trois-Rivières (CER-22-288-10.04) Research Ethics Boards have reviewed and approved this study.

### 2.2 Study design and setting

A retrospective cross-sectional analysis of medical files data was performed, using electronic health records of patients who attended for the first time the CIUSSS-MCQ’s outpatient neurosurgery clinic for a primary complaint of LBP between September 1st, 2018 (CRDS implementation date), and September 1st, 2021. The CIUSSS-MCQ was created following the fusion of regional public health and social establishments, including hospitals, community services and long-term facilities, and serves a population of more than 530,000 people, including 82.5% over the age of 18 (21). During the study period, the neurosurgery department was staffed by 5 neurosurgeons. CRDS’s referral form for neurosurgery services (see Supplementary File 1) assigns each patient a clinical priority code from A to E based on their clinical profile. Each priority code is associated with a specific delay to be observed between the referral being sent and the neurosurgery consultation (i.e., code A: ≤ 3 days, code B: ≤ 10 days, code C: ≤ 28 days, code D: ≤ 3 months, and code E: ≤ 12 months). As a prerequisite for the neurosurgery service, patients with LBP must also provide an imaging report dated less than 3 months prior to their surgical consultation.

### 2.3 Study population and patient selection

An administrative agent proceeded to the identification of potentially eligible medical files by searching the CRDS database based on listed reasons for referral. Inclusion criteria for eligible chart review included: [1] a documented reference to the CIUSSS-MCQ neurosurgery outpatient clinic; [2] a primary chief complaint that prompted consultation consistent with LBP; [3] an initial consult that took place between September 1st, 2018, and September 1st, 2021; and [4] included a patient that was 18 years or older at the time of the initial consult. Exclusion criteria were charts documenting follow-up visits only, and a primary chief complaint of unspecified spinal pain region. Two independent reviewers screened all potentially eligible medical files to confirm eligibility. A third reviewer was involved if consensus could not be reached. From the medical records meeting the inclusion criteria, a random selection of 500 files was made using block stratification to ensure representativeness of time periods (years), sexes and ages in the planned analyses. Those medical records were then subjected to data extraction. The sample size was based on existing literature which generally holds 10 charts per variable as an accepted norm to obtain results that are likely to be clinically useful (22).

### 2.4 Data collection

The medical record of each randomly selected patient was reviewed, and data were extracted from the neurosurgery outpatient consult request form, and from the consultation note. If more than one neurosurgery consult request form and consultation note were present in the medical file, only data related to the initial consultation were extracted. Data collection was performed using a standardized form with pre-set drop-down fields and free-texts boxes. Five patients meeting inclusion criteria were randomly selected and subsequently contacted by the neurosurgeon research team member (C.É.C) to obtain their consent for their records to be analyzed. This exploratory analysis has allowed to refine the form and confirm the data sets to be extracted. Four medical students were recruited and trained for data extraction. They were advised to extract only information related to the current episode of LBP. Accordingly, dates of clinical examinations performed were retrieved to ensure that they were related to the current episode. Whenever possible, the medical residents were asked to transcribe textually the written referral form and consultation note to avoid any interpretation of the data.

### 2.5 Data coding and outcomes

Table 1 presents the types and definitions of variables that were extracted to allow the drawing of a comprehensive portrait of patients being referred to the neurosurgery outpatient clinic. The extracted data were coded by four research assistants, using a codebook to ensure consistency. A training exercise was conducted with 15 medical files. Random verifications were subsequently performed by the main author (J.M.) throughout the extraction process to ensure reliability of the extracted data. To minimize subjectivity in codification in relation to personal theories about the study’s aims, data extractors were kept blinded to the study hypothesis (23, 24). When necessary, the neurosurgeon research team member (C.É.C) was consulted to provide information on standards of practice, and to clarify some medical terms and abbreviations. The Material and Social Deprivation Index (MSDI) was used as a proxy for lacking information on patients’ socioeconomic status in the CRDS database. The MSDI allows for a comprehensive assessment of social inequalities (25), by connecting area-based socioeconomic data to postal codes. The MSDI was carefully chosen due to its well-established associations with health status and deprivation, encompassing both material and social dimensions (26). The material dimension involves deprivation of the goods and conveniences that are part of modern life and marks the consequences of lack of material resources associated to low education, insecure job situation, and insufficient income. The social component refers to the composition and fragility of the social network (26, 27). For both dimensions, areas were ranked in quintiles, with quintile 1 being the most privileged and quintile 5 the most disadvantaged (25).

**Table 1:** Variables and outcomes.

Category	Extracted variable	Outcome
Sociodemographic data	1. Date of birth 2. Biological sex 3. City of residence, postal code, administrative region	1.1 Age 2.1 Male or Female 3.1 Material and Social Deprivation Index
Administrative data	4. Involvement of workers’ compensation board (CNESST) 5. Involvement of public automobile compensation board (SAAQ) 6. Date of referral Date of consult Priority code 7. Referring clinicians	4.1 Yes or No 5.1 Yes or No 6.1 Delay to neurosurgery consult 6.2 Pre-pandemic or pandemic period 6.3 A-B-C-D-E 6.4 Compliance with the priority code 7.1 Primary care referral, medical specialist, emergency unit
Clinical data	8. Medical history 9. Presence of red flags 10. Pain localization and dominance 11. Pain status 12. Pain duration 13. Symptoms progression 14. Presence of neurological deficits 15. Diagnosis from the neurosurgeons	8.1 Type and number of comorbidities 8.2 Pain medication 8.3 History of spinal surgery 8.4 Smoking status 9.1 i.e., bladder and bowel dysfunction, saddle anesthesia, fever, chills, history of trauma 10.1 Back, leg(s) or both 11.1 Intermittent, constant 12.1 Acute (< 4 w), Subacute (4-12w) or chronic (> 12 w) 13.1 Stable, aggravation, improvement 14.1 Sensory, motor, reflex deficits 14.2 Presence of pathological reflexes (i.e., clonus, Babinski sign) 15.1 Type of LBP (i.e., non-specific LBP, radicular syndrome, specific LBP)
Care trajectories data	16. Use of advance imaging and diagnostic testing for LBP prior to the neurosurgery consult 17. Use of conservative care prior to the neurosurgery consult	16.1 Type of diagnostic test 17.1 Type of health professional seen
Consultation outcomes	18. Recommended management strategy	18.1 Proportion of discharges from the neurosurgery service 18.2 Recommendations upon discharge 18.3 Proportion of referrals made to another service 18.4 FUP interventions 18.5 Surgical indication

FUP, Follow-up; LBP, Low back pain; w, weeks.

#### 2.5.1 Referral appropriateness

For the 500 medical files randomly selected, two clinical researchers (J.M, A.A.M.) independently judged the appropriateness of referrals made to the neurosurgery department. Referral appropriateness was judged by considering 3 specific components: [1] the compliance with CRDS’s referral form criteria (Supplementary Files 1, 2), such as (a) the presence of a painful or sensory-motor radiculopathy OR neurogenic claudication with either (i) severe symptoms and functional limitations >8 weeks or (ii) moderate chronic symptoms >8 weeks OR (b) the presence of an isolated LBP with structural abnormality; [2] the patient’s clinical profile; and [3] the patient’s care trajectory. From a clinical perspective, referrals were considered inappropriate if one or more of the following criteria was present: [1] pain pattern consistent with non-specific LBP; [2] acute pain episode or non-progressing or slowly progressing symptoms; and [3] absence of objective neurological symptoms.

Patients’ care trajectories were deemed inappropriate if diagnostic tests and conservative treatment options had not been fully exhausted (guideline concordant care for 6–10 weeks) prior to referral for specialized care. These criteria were based on evidence-based guidelines for the management of LBP (28–32) and on factors known to be associated with poor surgical outcomes (33–36). Researchers met to discuss disagreements and to reach consensus. A third researcher (M.D.) was involved if consensus could not be reached.

### 2.6 Data analysis

The Shapiro–Wilk normality test was performed for all continuous variables to determine data distribution. Patient characteristics, care trajectories and consultation outcomes were summarized using frequency distributions for categorical variables, and means, medians and standard deviations for continuous variables. For between groups comparisons (i.e., appropriate vs. inappropriate referrals; pre-pandemic vs. pandemic period), Mann–Whitney test and independent Student *t* test were used for continuous variables and the chi-square test with pairwise *Z*-tests were used for categorical variables. After comparing the characteristics of appropriate and inappropriate referrals, variables that differed significantly between groups were introduced in the binomial logistic regression model using the enter method (i.e., all variables were entered in the model in a single step) to determine whether they predicted the appropriateness of referrals made to the neurosurgery service. Odds ratios (OR) and 95% confidence intervals (95% CI) were calculated and reported for each included variable. Variables that were missing in more than 20.0% of our sample were not included in logistic regression analyses. All statistical analyses were performed using IBM SPSS Statistics for Windows, Version 18.0.0 (Armonk, NY: IBM Corp.) and considered p values <0.05 statistically significant.

## 3 Results

### 3.1 Availability of data

A total of 2,965 medical records met the inclusion criteria, of which 500 were randomly selected. Following a preliminary analysis of our random sample, 118 files had to be replaced by a random selection, since at least one of the three components used to judge the appropriateness of referrals lacked information. Socio-demographic data were available in 100% of selected files. For the MSDI, 10.0% of patients had zip codes for which no corresponding deprivation index value was available. As for administrative data, the clinical priority code was not documented for 16.6% of patients. Several data pertaining to patients’ clinical profiles were missing. Pain dominance and pain status were reported for 31.6 and 9.0% of the study sample, respectively, while back or leg pain intensity was only reported for 2.0% of cases. The presence or absence of red flags were not documented for 45.6% of patients. For 13.6% of the sample, none of the neurological examination components were described. The progression of symptoms and patients’ medical history were not documented in 45.8 and 41.6%, respectively. While diagnostic tests performed prior to the neurosurgery consultation were reported for almost all patients, conservative treatments received were not documented for 38.2% of the sample.

### 3.2 Specific objective 1

The following sections outline the characteristics documented in medical files of patients referred to the CIUSSS-MCQ’s neurosurgery service for a primary complaint of LBP. Proportions for each characteristic have been computed based on the total number of selected files (*N* = 500). The percentage of missing values for each variable is detailed in corresponding Tables 2–6.

**Table 2:** Sociodemographic characteristics.

Characteristics	*N* (%) or mean ± SD	Missing value, *n* (%)
Age (years)	60.06 ± 15.28	-
Biological sex		
Male	262 (52.4)	-
Female	238 (47.6)	
Administrative region		
Mauricie	279 (55.8)	-
Centre-du-Québec	154 (30.8)	
Lanaudière	44 (8.8)	
Other	23 (4.6)	
Material deprivation index		
5	141 (28.2)	50 (10.0)
4	122 (24.4)	
3	91 (18.2)	
2	53 (10.6)	
1	43 (8.6)	
Social deprivation index		
5	97 (19.4)	50 (10.0)
4	62 (12.4)	
3	98 (19.6)	
2	120 (24.0)	
1	73 (14.6)	

SD, Standard deviation.

**Table 3:** Administrative data.

Characteristics	*N* (%)	Missing value, *n* (%)
Involvement of compensation boards		
CNESST (yes)	34 (6.8)	-
SAAQ (yes)	4 (0.8)	
Consultation period		
Pre-pandemic (< 2020-03-13)	306 (61.2)	-
Pandemic (≥ 2020-03-13)	194 (38.8)	
Delay to the neurosurgery consult (days)	Median: 50.50 IQR: 75.75	-
Priority code		
A (**≤** 3 days)	5 (1.0)	83 (16.6)
B (**≤** 10 days)	10 (2.0)	
C (**≤** 28 days)	47 (9.4)	
D (**≤** 3 months)	284 (56.8)	
E (**≤** 12 months)	71 (14.2)	
Compliance with priority code		
Yes	289 (57.8)	83 (16.6)
No	128 (25.6)	
Referring clinicians		
General physician	442 (88.4)	-
Physiatrist	13 (2.6)	
Orthopedist	8 (3.6)	
Neurologist	5 (1.0)	
Other	32 (6.4)	

CNESST, Commission des normes de l’équité, de la santé et de la sécurité du travail; IQR, Interquartile range; SAAQ, Société de l’assurance automobile du Québec.

**Table 4:** Clinical profiles.

Characteristics	*N* (%) or mean ± SD	Missing value, *n* (%)
Presence of red flags		
Yes	39 (7.8)	228 (45.6)
No	233 (46.6)	
Neurological status		
Motor deficits	92 (18.4)	100 (20.0)
Sensory deficits	144 (28.8)	157 (31.4)
Reflexes deficits	109 (21.8)	215 (43.0)
Pathological reflexes	10 (2.0)	391 (78.2)
Pain location		
Back (yes)	403 (80.6)	78 (15.6)
Leg(s) (yes)	407 (81.4)	35 (7.0)
Pain dominance		
Back	97 (19.4)	342 (68.4)
Leg(s)	61 (12.2)	
Pain status		
Intermittent	33 (6.6)	455 (91.0)
Constant	12 (2.4)	
Pain intensity		
Back pain	7.0 ± 2.7	491 (98.2)
Leg(s) pain	5.4 ± 3.6	495 (99.0)
Pain duration		
Acute (< 4 weeks)	4 (0.8)	33 (6.6)
Subacute (4–12 weeks)	40 (8.0)	
Chronic (> 12 weeks)	423 (84.6)	
Progression of symptoms		
Stable	34 (6.8)	229 (45.8)
Aggravation	129 (25.8)	
Improvement	108 (21.6)	
Smoking status		
Smoker	59 (11.8)	353 (70.6)
Previous history of spinal surgery		
Yes	87 (17.4)	205 (41.0)
Usage of pain medication		
Yes	247 (49.4)	205 (41.0)
Comorbidities (number)		
0–1	115 (23.0)	208 (41.6)
Multimorbidity (**≥**2)	177 (35.4)	
Comorbidities (type)		
Cardiovascular (e.g., HTA)	155 (31.0)	208 (41.6)
Endocrine (e.g., DB, Hypo/Hyperthyroidism)	97 (19.4)	
Urogenital (e.g., renal failure)	50 (10.0)	
Pulmonary (e.g., COPD)	47 (9.4)	
Gastrointestinal (e.g., IBS)	46 (9.2)	
Other	145 (29.0)	
Neurosurgeons’ diagnoses		
Non-specific LBP	179 (35.8)	-
Radicular syndrome	304 (60.8)	
Specific LBP	9 (1.8)	
Other causes	8 (1.6)	

COPD, Chronic obstructive pulmonary disease; DB, Diabetes; HTA, Hypertension; IBS, Irritable bowel syndrome.

**Table 5:** History of diagnostic testing and conservative treatment.

Characteristics	*N* (%)	Missing value, *n* (%)
Diagnostic tests prior to the neurosurgery consult		
Yes	487 (97.4)	13 (2.6)
Diagnostic tests (type)		
MRI	387 (77.4)	13 (2.6)
CT-scan	163 (32.6)	
X-rays	45 (9.0)	
EMG	28 (5.6)	
Other	3 (0.6)	
Conservative care prior to the neurosurgery consult		
Yes	282 (56.4)	191 (38.2)
No	32 (6.4)	
Type of treatment		
Anesthetic injections	209 (41.8)	235 (47.0)
Physiotherapy	128 (25.6)	321 (64.2)
Chiropractic	17 (3.4)	483 (96.6)
Massage therapy	8 (1.6)	492 (98.4)
Occupational therapy	6 (1.2)	-

CT-scan, Tomodensitometry; EMG, Electromyography; MRI, Magnetic resonance imaging; X-rays, Radiographs.

**Table 6:** Outcomes of neurosurgery consultations.

Characteristics	*N* (%)	Missing value, *n* (%)
Recommended management strategy		
A. Discharge from the neurosurgery service (total)	221 (44.2)	3 (0.6)
B. Non-surgically managed by neurosurgeons	199 (39.8)	-
C. Received a clear or relative surgical indication	112 (22.4)	25 (5.0)
A. Recommendations upon discharge (*N* = 221)		
No recommendations	92 (18.4)	-
Anesthetic injections	56 (11.2)	-
Conservative care	27 (5.4)	-
Combination	33 (6.6)	-
Referral to another health professional	16 (3.2)	-
B. FUP interventions (*N* = 199)		
Imaging referral with FUP	53 (10.6)	-
Referral for anesthetic injections with FUP	67 (13.4)	-
Referral for conservative care with FUP	13 (2.6)	-
Referral for treatment combination with FUP	36 (7.2)	-
Referral to another specialist with FUP	5 (1.0)	-
Wait and see	25 (5.0)	-
C. Surgical indication (*N* = 112)		
Clear indication	62 (12.4)	25 (5.0)
Relative indication^a^	50 (10.0)	
Reluctant or refused to undergo surgery despite surgical indication	21(18.8)	-

FUP, Follow-up; LBP, Low back pain. ^a^Relative surgical indication: Potential surgical indication if symptoms persist or worsen after conservative treatment or for clinically stable patients with persistent disabilities.

#### 3.2.1 Sociodemographic data

Table 2 presents the sociodemographic characteristics of the 500 patients included in the study. The mean age was 60.06 ± 15.28 years at the time of the initial neurosurgery consultation, and 52.4% of patients were male. More than half (55.8%) of patients were living in the administrative region of Mauricie, and 30.8% in the Centre-du-Québec, while 13.4% of patients originated from regions beyond the territory served by the CIUSSS-MCQ. More than half of our study sample (52.6%) was classified in the 4th and 5th quintiles for material deprivation (i.e., most disadvantaged categories), while 8.6% of patients fell into the first quintile (i.e., most privileged category) for this component. As for social deprivation, 31.8% of patients were ranked in the 4th and 5th quintiles, while only 14.6% fell into the most privileged category.

#### 3.2.2 Administrative data

Administrative data extracted from patients’ medical records are presented in Table 3. In the study sample, 61.2% of initial consultation took place before the COVID-19 pandemic outbreak in Canada. A 63.4% decrease in neurosurgery referrals for LBP was observed between the pre-pandemic and the pandemic period. Most of the neurosurgery outpatient consultation requests came from primary care physicians (88.4%), followed by medical specialists such as orthopedists (3.6%), physiatrists (2.6%), and neurologists (1.0%). The clinical priority code “D” (i.e., ≤ 3 months) was attributed to over half (56.8%) of referred patients, while the priority code “E” (i.e., ≤ 12 months) was attributed to 14.2% of the sample, and “C” (i.e., ≤ 28 days) to 9.4%. Slightly more than a quarter (25.6%) of reviewed consultations were not carried out within the timeframe prescribed by the priority code. The percentage of consultations that failed to take place within the recommended timeframe was significantly higher during the pandemic period (35.1%) than during the pre-pandemic period (19.6%). Only 7.6% of patients were entitled to compensation from the *Commission des normes, de l’équité, de la santé et de la sécurité au travail* (workers’ compensation board) and the *Société de l’assurance automobile du Québec* (public automobile compensation board) in relation to their LBP.

#### 3.2.3 Clinical profiles

Patients’ clinical characteristics are detailed in Table 4. In 19.4% of medical records, patients suffered from dominant back pain, and 6.6% exhibited intermittent symptoms. Red flags were identified in 7.8% of patients. Most patients (84.6%) were experiencing chronic pain (> 12 weeks) at the time of their initial neurosurgery consultation, 28.8% had objective sensory deficits, 21.8% showed asymmetrically diminished tendon reflexes, and 18.4% had objective motor deficits. Over a quarter (25.8%) of patients presented to the neurosurgery service with deteriorating symptoms, with 21.6% of patients experiencing improvement in symptoms since the neurosurgery referral. A review of patients’ medical history revealed that 17.4% had a previous history of lumbar surgery, 49.4% were using pain medication, and 11.8% were smokers. In 35.4% of cases, patients were classified as multimorbid, presenting two or more comorbidities (37), the most frequent being cardiovascular diseases (31.0%), endocrine diseases (19.4%) and urogenital disorders (10.0%) As for neurosurgeons’ diagnoses, 60.8% of patients were diagnosed with a radicular syndrome, which included pain patterns consistent with lumbar radiculopathy, lumbar spinal stenosis, and neurogenic claudication. The diagnosis of non-specific LBP was attributed to 35.8% of patients included in the analysis, with the remaining suffering from specific LBP (1.8%) or experiencing symptoms unrelated to a lumbar spine disorder (1.6%) (e.g., cervical myelopathy, peripheral joints disorders, vascular disorders).

#### 3.2.4 Care trajectories data

Detailed information regarding patients’ care trajectories is provided in Table 5. Nearly all (97.0%) patients were referred for further diagnostic testing prior to their initial neurosurgery consultation, with MRI (77.4%) and CT-scan (32.6%), being the most frequently prescribed imaging procedures. Over half (56.4%) of patients received conservative treatments prior to the neurosurgery consultation, including 41.8% who received anesthetic injections, 25.6% who underwent physiotherapy treatments, and 3.4% who had seen a chiropractor.

#### 3.2.5 Consultation outcomes

Neurosurgery consultations’ outcomes are provided in Table 6. Upon initial assessment, a clear or relative (i.e., potential surgical indication if symptoms persist or worsen after conservative treatment or for clinically stable patients with persistent disabilities) spinal surgery indication was documented in 22.4% of patients. Forty-four percent of patients were discharged from the neurosurgery service, including 18.4% for whom no recommendations were documented, 11.2% who were referred for anesthetic injections, 5.4% for conservative treatments, and 3.2% to another health care professional. For patients non-surgically managed by neurosurgeons (39.8%), 13.4% were also referred for anesthetic injections, 10.6% for advanced imaging, and 7.2% for a combination of conservative treatments, advanced imaging, and anesthetic injections.

### 3.3 Specific objective 2

#### 3.3.1 Referral appropriateness

In 80.4% of cases, referrals made to the neurosurgery department for a primary complaint of LBP during the study period were deemed inappropriate. The proportion of inappropriate referrals was similar during the pandemic period when compared to the pre-pandemic period (*p* = 0.467). Table 7 details the number of cases that failed to meet the CRDS, clinical and care trajectories criteria for a neurosurgery consultation. Regarding the CRDS criteria, 6.2% of patients presented to their initial neurosurgery consultation without the appropriate imaging (i.e., MRI or CT) or with imaging performed over 3 months prior to the consultation. For 9.7% of the study sample, patients were deemed inappropriately referred as they exhibited symptoms of acute or subacute duration. From a clinical perspective, 43.0% of patients presented pain patterns consistent with non-specific LBP, and the absence of objective neurological symptoms was reported in 28.1% of cases. Stable or improving symptoms were documented in 32.6% of patients. As for patients’ care trajectories, the absence of a conservative care trial prior to the neurosurgery consultation was documented for 7.0% of patients.

**Table 7:** Referral appropriateness.

Characteristics	*N* (%)	Missing value, *n* (%)
Appropriateness		
Inappropriate	402 (80.4)	2 (0.4)
Non-compliance with CRDS referral form criteria		
Absence of appropriate or up to date (< 3 months) imaging	25 (6.2)	13 (2.6)
Acute (< 4 weeks) or subacute (4–12 weeks) pain episode	39 (9.7)	33 (6.6)
Non-compliance with clinical criteria		
Pain pattern consistent with non-specific LBP	173 (43.0)	-
Stable or improving symptoms	131 (32.6)	229 (45.8)
Absence of objective neurological symptoms	113 (28.1)	68 (13.6)
Non-compliance with care trajectories criteria		
No conservative care trial prior to the neurosurgery referral	28 (7.0)	191 (38.2)
Conservative treatment options have not been exhausted when indicated	219 (56.2)	

LBP, Low back pain.

### 3.4 Specific objective 3

#### 3.4.1 Comparison of appropriate and inappropriate referrals

Socio-demographic profiles and the median delay to the neurosurgery consultation were comparable between inappropriately and appropriately referred patients (*p* > 0.05), except for the social deprivation index, for which a higher proportion of patients ranked in the most privileged quintile was found in the appropriate referrals group compared to the inappropriate referrals group (23.5% vs. 14.5%). As for patients’ clinical profiles, we found a significantly higher proportion of patients suffering from dominant back pain in the inappropriate referrals group (22.9%) compared to the appropriate referrals group (4.2%). Progression of symptoms differed significantly between groups as the inappropriate referrals group had significantly higher proportion of patients symptomatically improved (24.9% vs. 8.4%), while the appropriate referrals group included a higher proportion of patients with deteriorating symptoms (33.7% vs. 23.6%). Inappropriate referrals group presented significantly higher proportions of patients with no objective sensory deficits (42.5% vs. 28.4%) and no objective motor deficits (64.7% vs. 47.4%). A pain pattern consistent with non-specific LBP has been described in 43.0% of patients deemed inappropriately referred, while only 5.3% of patients diagnosed with non-specific LBP were deemed appropriately referred (i.e., patients for whom the predominant clinical profile was consistent with non-specific LBP but who presented intermittent neurological symptoms requiring follow-up). In regression analysis, only two variables could be investigated for inappropriate referencing since the other independent variables with a p value <0.05 exceeded the pre-established 20.0% cut-off for missing values. The absence of motor deficits (*p* < 0.001; OR 2.91; 95% CI 1.62–5.2) and being ranked in the third quintile for social deprivation (*p* < 0.039; OR 2.75; 95% CI 1.05–7.18) both appeared as predictors of inappropriate referencing to the neurosurgery service for LBP. Results of regression analyses are detailed in Supplementary File 3.

## 4 Discussion

This study aimed to draw a comprehensive portrait of patients referred to specialized care for LBP, to evaluate the appropriateness of referrals, and to identify the characteristics that differentiated inappropriate from appropriate referrals.

The study sample comprised a random selection of 500 patients, with a mean age of 60 years old, reflecting the age-related increase in prevalence of degenerative lumbar conditions, which account for a significant proportion of cases deemed likely to require surgical consultation (38). Our sample included similar proportions of males and females, although the overall mean prevalence of LBP is known to be higher among females than males across all age groups (39). Referrals for LBP decreased significantly between the pre-pandemic and the pandemic period, which might suggest that patients were avoiding healthcare services unless they had critical symptoms (40, 41). Most referrals (88.4%) made during the study period came from general physicians, which is consistent with Canada’s healthcare delivery model, whereby patients must first be seen by a primary care provider before they can access specialized care (1). The small but noteworthy proportion (13.4%) of referrals received from administrative regions not served by the CIUSSS-MCQ may be explained, though not exclusively, by long waiting lists affecting surrounding neurosurgery services, which may prompt physicians to send multiple referrals to maximize the patient’s likelihood of a timely consultation (42). Nonetheless, more than 25.0% of CIUSSS-MCQ’s neurosurgery consultations for LBP during the study period were not carried out within the expected delay. This percentage is, however, lower than the provincial average which was reported at 33.0% for the year 2021–2022 (5).

Evidence-based guidelines recommend diagnostic triage to classify patients into one of three type of LBP (i.e., non-specific LBP, radicular syndrome, or specific LBP) and suggest management strategies tailored to each of these categories (28, 30–32, 43). As an example, patients diagnosed with persistent (> 4–6 months) non-specific LBP should be provided with structured patient education and multimodal conservative care, whereas a diagnosis of persistent lumbar radiculopathy usually calls for referral for further investigation (32). Diagnostic accuracy is therefore considered crucial to determine whether the patient’s condition warrants further investigation or a specialist referral. A recent scoping review of systematic reviews (44) identified several clinical features with appropriate diagnostic value, and therefore, suitable for use in primary care settings for the diagnosis of LBP. Overall, dominant site of pain, pain distribution, aggravating and relieving factors, indicators of underlying spinal pathology and the presence of neurological signs should all be assessed or questioned when evaluating LBP patients (44). Various clinical characteristics are also known to predict response to surgical treatment for LBP, such as the level of disability, baseline leg pain intensity, smoking status, psychological complaints, frailty status and comorbidities, previous spinal surgeries, and patient expectations of treatment outcomes (33–36). Surprisingly, many of these variables were inconsistently documented in the CRDS referral form or in the consultation note, thus preventing the establishment of a comprehensive portrait of patients referred to the neurosurgery service for LBP. It remains to be determined whether this reflects a lack of documentation, or if these variables are being overlooked in clinical decision-making in favor of other clinical characteristics that would further inform clinical decisions. These findings are however consistent with a previous review of Canadian spine surgeons referrals (15), which demonstrated that many factors used in surgical decision-making were not routinely documented, and that most referrals to spine surgeons lacked adequate clinical information for triage. Nevertheless, we were able to determine that most of the sample suffered from chronic pain (84.6%), had undergone diagnostic testing prior to their neurosurgery consultation (97.4%), and had been diagnosed with a radicular syndrome (60.8%). Interestingly, of the 271 patients for whom progression of symptoms was documented, 60.1% exhibited stable or improving symptoms at the time of their initial neurosurgery consultation, potentially reflecting the fluctuating and self-limiting nature of LBP (45–48). These findings also support evidence-based guidelines recommendations, which advocate that patients with LBP should undergo a reasonable conservative care trial before contemplating referral to a medical specialist. Although considered a prerequisite for surgical consultation for LBP, it could not be determined for almost 40.0% of patients whether they had received appropriate conservative treatments before seeking neurosurgery services.

Our analysis revealed that 80.4% of referrals made to the CIUSSS-MCQ’s outpatient neurosurgery clinic for LBP during the study period were deemed inappropriate. Our study findings are in line with other Canadian studies, which have previously reported that between 62.0–85% of referrals to spine surgeons for LBP were inappropriate (16–19, 49). Of the 500 medical files reviewed, only 20.4% of patients were identified as surgical candidates, and of these, 18.8% were reluctant or explicitly refused any surgical options. Interestingly, a study by Mayman et al. (18) also reported a small but significant proportion (13.0%) of referred LBP patients who expressed reluctance to undergo surgery regardless of its indication. This reinforces the importance of promoting shared-decision making, in which patients and providers work together to find a mutually agreed-upon treatment plan, as this approach is known to improve quality of care delivery, and to reduce the overuse of surgical procedures (50, 51). Over 40.0% of LBP patients referred to the neurosurgery service during the study period were discharged upon initial assessment, most of them after being advised to initiate or pursue conservative treatments or after being referred for anesthetic injections. Of the 199 patients non-surgically managed by the neurosurgeons, 87.4% were referred for further diagnostic or therapeutic interventions before undergoing further neurosurgical follow-up, suggesting that non-surgical options had not been fully exhausted for these patients. The high proportion of patients referred for anesthetic injections, commonly seen as a therapeutic modality, could also be explained by evidence supporting the ability of spinal injections to predict surgical outcomes (52, 53). In a retrospective analysis of medical files data from patients with LBP referred to the neurosurgery service of 3 European hospitals, Debono et al. (54) raised a similar issue, identifying a significant proportion of patients inappropriately referred who had not properly been treated before being referred to the neurosurgery service or whose imaging tests were incomplete.

Inappropriate referrals were characterized by higher proportion of patients symptomatically improved, presenting a back-dominant chief complaint, exhibiting no objective neurological symptoms, and diagnosed with non-specific LBP. The multivariate analysis also suggested that the absence of motor deficits was associated with a nearly three-fold increase in the odds of being inappropriately referred. In a retrospective review of spine referrals sent to a group of ten neurosurgeons in Edmonton, Canada, over a 3-year period (2007–2009), Deis and Findlay (49) reached similar conclusions, finding that most inappropriate referrals were based on no mention of leg symptoms or signs of neurological deficits rather than on the lack of concordance between lumbar spine imaging findings and the patient’s clinical profile. Debono et al. (54) reported similar results, noting that only 5.1% of inappropriate referrals to the neurosurgery service for LBP were attributed to radioclinical discordance. Additionally, a retrospective study of new referrals for LBP to the neurosurgery service of an Australian public hospital found that there was no significant association between MRI findings and the likelihood of undergoing surgery (55). This is of particular interest as it calls into question the value of imaging findings as a component that can serve to justify a referral to the neurosurgery service. In the present study, imaging results were more frequently reported by referring clinicians than any other type of clinical information, while documentation of pain predominance, level of disability, and neurological findings was often overlooked. However, it is known that up to 90.0% of patients over the age of 50 will have evidence of age-related degenerative changes without definite nerve root compression (56) on their lumbar spine MRI or CT reports, and that these imaging findings may even be seen in asymptomatic individuals (57). Overreliance on imaging procedures could, however, be explained by the constraints imposed by the CRDS’s referral form, which explicitly requires that patients seeking referral for the neurosurgery service provide an imaging report dated less than 3 months. In addition to potentially increasing the importance placed on imaging results, this requirement also raises a potential issue in the referral process. Indeed, given the extended wait times associated with priority codes D and E, it appears likely that patients assigned to theses codes may struggle to comply with this criterion, thus questioning its relevance. This reinforces evidence-based recommendations according to which decision-making should be informed first and foremost by the patients’ clinical profile, and that routine imaging should be avoided and used only in the presence of potential indication for surgical consultation. Furthermore, this also underlines the need to reinforce continuing medical education for primary care providers, focusing on the clinical criteria justifying spinal imaging or referral to a spine surgeon.

Several strategies to improve the appropriateness of referrals to spine surgeons for LBP have been studied. Though currently not implemented on Quebec’s territory, triage interventions have shown promising results in managing LBP patients. Two retrospective analyzes of new outpatient referrals for LBP (17, 19) showed that a multidisciplinary triage process led by MSK experts, also known as the *Saskatchewan Spine Pathway*, significantly reduced MRI utilization and inappropriate referrals to spine surgeons compared to the conventional referral process (i.e., patients referred directly by primary physicians). A retrospective audit of data from three Australian public hospital emergency departments (ED) showed that patients with LBP managed by advanced MSK physiotherapists had shorter ED wait times and length of stay and were more effectively discharge compared with patients seen by ED doctors and nurse practitioners (58). Another retrospective review of all new patients visits to eight orthopedic surgeons at a large academic hospital in New York also revealed that patients triaged by MSK healthcare providers were more likely to undergo surgery (59). Isolated surgical decision-making is known to result in suboptimal treatment recommendations (60). Although more research is needed in the field, multidisciplinary approaches, drawing on MSK healthcare providers expertise, seem more likely to improve the selection of surgical candidates, while providing other patients with appropriate nonoperative treatment options. In an in-dept review and critical analysis, Foster et al. (61) evoke compelling arguments for considering other models of first-contact MSK care, notably ones whose point of entry in healthcare are MSK experts such as physiotherapists or chiropractors. Indeed, evidence suggest that access to primary care services run by MSK specialists is associated with higher improvements in patients’ outcomes, a reduction in healthcare use, and with fewer days off work related to back pain (62, 63).

### 4.1 Strengths and limitations

This study stands out for its comprehensive examination of patients referred to specialized care for LBP, providing a detailed understanding of clinical profiles and referral patterns of patients most likely to benefit from surgical consultation. This is also the first study to explore the appropriateness of referrals to specialized care for LBP in the context of a publicly funded healthcare system operating a CWL, offering insight into the challenges within the existing healthcare infrastructure. Despite a rigorous methodology, this study has some limitations. Although the study provides a detailed description of patients referred to specialized care for LBP and examines referrals appropriateness, its generalizability is limited as it was performed in a single administrative region. The CIUSSS-MCQ covers a vast territory (64) and provides services to diverse populations, including remote and indigenous communities (65). These populations may face additional challenges in terms of availability and accessibility of healthcare resources. Consequently, these results may not be generalizable to large urban centers. The study findings are also limited by the quality of data collected. Indeed, key information was often not documented in medical files, and most information was hand-written, which may have hampered data interpretation. Considering that some medical files were either incomplete, or even had to be excluded for lack of information, it is important to acknowledge the potential presence of selection bias, as the characteristics of the patients who were not included in the analysis may differed from those in our study sample. Several variables could also not be included in the binomial regression model, most of them known for their appropriate diagnostic value or their ability to predict surgical outcomes. Further studies are needed to determine whether these would predict the appropriateness of referrals made to the neurosurgery service. Using a complementary qualitative approach could help fill the gaps in chart documentation and provide a better understanding of both the factors that prompted referrals to medical specialists for LBP, and of the criteria considered by neurosurgeons when selecting the appropriate management strategy.

## 5 Conclusion

Through a comprehensive analysis of socio-demographic, administrative, and clinical data, this retrospective chart review echoes the findings of previous studies, suggesting that a significant proportion of patients are inappropriately referred to specialized care for a primary complaint of LBP. The generalizability of the study findings is however limited, as the data were sourced from a single administrative region, and several incomplete medical files had to be excluded from the analysis. Back-dominant chief complaints, the absence of objective neurological deficits, and a non-specific LBP diagnosis were identified as characteristic features of inappropriate referrals. While evidence-based guidelines advocate for a thorough clinical assessment for effective diagnostic triage, this analysis unveiled the absence of key clinical information in referral forms and consultation notes, emphasizing the urgent need to enhance documentation practices. Reliance on imaging findings as a criterion for neurosurgery referral was also questioned, as it seemed to further influence clinical decisions to the detriment of factors known to predict surgical outcomes. This study also suggests that an increased focus should be placed on multidisciplinary approaches that enable LBP patients to get a timely access to the appropriate healthcare provider. Collaborative efforts involving MSK experts in triaging patients with LBP have been shown to improve the selection of surgical candidates, while precluding the use of ineffective diagnostic and therapeutic approaches. However, further research is needed to assess the impact of those innovative strategies within Quebec’s healthcare delivery model.

## Data availability statement

The data analyzed in this study is subject to the following licenses/restrictions: the raw data supporting the conclusions of this article will be made available by the authors, without undue reservation, upon reasonable request. Requests to access these datasets should be directed to JM, janny.mathieu@uqtr.ca.

## Ethics statement

The studies involving humans were approved by the Centre intégré universitaire de santé et de services sociaux de la Mauricie-et-du-Centre-du-Québec (CER-2022–600-838) and the Université du Québec à Trois-Rivières (CER-22-288-10.04) Research Ethics Boards. The studies were conducted in accordance with the local legislation and institutional requirements. Written informed consent for participation was not required from the participants or the participants’ legal guardians/next of kin in accordance with the national legislation and institutional requirements.

## Author contributions

JM: Formal analysis, Methodology, Writing – original draft, Conceptualization, Project administration, Validation, Visualization. M-ÈR: Investigation, Writing – review & editing. C-ÉC: Validation, Writing – review & editing. MD: Conceptualization, Supervision, Writing – review & editing. A-AM: Conceptualization, Funding acquisition, Supervision, Writing – review & editing, Validation.
