# Supplementary material for: Appropriateness of specialized care referrals for LBP: a cross-sectional analysis
Source: Front Med (Lausanne). 2024 Jan 5;10:1292481. doi: 10.3389/fmed.2023.1292481 (PMC10797061; doi:10.3389/fmed.2023.1292481)
Supplement: Supplementary file 1 [file Data_Sheet_1.pdf]

Note to readers: The 2018 CRDS form was used to judge referral appropriateness, considering these referrals were made between September 1, 2018 and September 1, 2021. As the 2018 version of the form is not available in English, we provide the 2022 English version for ease of understanding. Note that in the updated version, the imaging report must now be dated less than 12 months prior to the neurosurgery consultation, whereas it had to be dated less than 3 months in the 2018 version. Other criteria remain unchanged.

Santé et Services sociaux Québec

DT9429

Nom et prénom de l'usager	
N° d'assurance maladie	Expiration Année Mois
Nom et prénom du parent	
Ind.rég. N° téléphone	Ind. rég. N° téléphone (autre)
Adresse	
	Code postal

CONSULTATION EN NEUROCHIRURGIE ADULTE

Attention :

1- Pour les priorités A et B (voir verso), communiquer directement avec le neurochirurgien de garde.

2- Aviser l'usager d'apporter une copie CD-ROM des imageries pour le rendez-vous avec le spécialiste.

Raison de consultation

Échelle de priorité clinique : A : ≤ 3 jrs B : ≤ 10 jrs C : ≤ 28 jrs D : ≤ 3 mois E : ≤ 12 mois

Section	Raison	Priorité
Rachis cervico-dorsal	☐ Myélopathie compressive avec symptômes > 8 semaines *(Prérequis : rapport d'IRM < 3 mois et autres rapports d'investigation)*	C
Rachis cervico-dorsal	Radiculopathie douloureuse ou sensitivo-motrice *(Prérequis : rapport d'IRM < 3 mois et autres rapports d'investigation)* — ☐ Avec symptômes sévères et limitation fonctionnelle (AVD/AVQ) > 8 semaines	D
Rachis cervico-dorsal	Radiculopathie douloureuse ou sensitivo-motrice — ☐ Avec symptômes modérés chroniques > 8 semaines	E
Rachis cervico-dorsal	☐ Cervicalgie/dorsalgie **avec instabilité anatomique**, démontrée à l'imagerie, sans atteinte médullaire ou radiculaire *(Prérequis : rapport d'imagerie et autres rapports d'investigation)*	D
Rachis lombo sacré	Radiculopathie douloureuse ou sensitivo-motrice ou claudication neurogénique *(Prérequis : rapport d'IRM < 3 mois et autres rapports d'investigation)* — ☐ Avec symptômes sévères et limitation fonctionnelle (AVD/AVQ) > 8 semaines	D
Rachis lombo sacré	Radiculopathie douloureuse ou sensitivo-motrice ou claudication neurogénique — ☐ Avec symptômes modérés chroniques > 8 semaines	E
Rachis lombo sacré	☐ Lombalgie isolée sans atteinte radiculaire avec anomalie structurelle (scoliose, spondylolyse, spondylolisthésis) *(Prérequis : rapport d'IRM < 3 mois et autres rapports d'investigation)*	E
Tumeur	☐ Tumeurs extracérébrales : méningiome, neurinome, schwannome, tumeur hypophysaire, base ou voûte du crâne *(Prérequis : rapport d'IRM ou TDM et autres rapports d'investigation)*	D
Vasculaire	☐ Anévrisme cérébral, malformation artério-veineuse fistule durale, angiome caverneux (sans hémorragie) *(Prérequis : rapport d'IRM ou TDM et autres rapports d'investigation)*	D
Vasculaire	☐ Sténose carotidienne asymptomatique (≥ 70 %) *(Prérequis : rapport d'imagerie et autres rapports d'investigation)*	D
Fonctionnel	☐ Névralgie cranio-faciale (ex. : trijumeau) réfractaire au traitement médical *(Prérequis : rapport d'IRM et autres rapports d'investigation)*	D
Fonctionnel	☐ Neuromodulation pour syndrome douloureux chronique ou pour spasticité *(Prérequis : rapports d'investigation)*	E
Crânien divers	☐ Hydrocéphalie chronique ou à pression normale *(Prérequis : rapport d'imagerie et autres rapports d'investigation)*	D
Crânien divers	☐ Kyste intracrânien (ex. : arachnoïdien, glande pinéale) *(Prérequis : rapport d'IRM ou TDM et autres rapports d'investigation)*	E
Crânien divers	☐ Lésion osseuse crânienne d'aspect bénin (ex. : kyste, malformation osseuse) *(Prérequis : rapport d'IRM ou TDM et autres rapports d'investigation)*	E
Nerfs périphériques	Neuropathie compressive (ex. : tunnel carpien ou canal cubital) *(Prérequis : EMG < 1 an et autres rapports d'investigation)* — ☐ Avec atteinte motrice	D
Nerfs périphériques	Neuropathie compressive — ☐ Sans atteinte motrice	E
Nerfs périphériques	☐ Tumeur d'un nerf périphérique *(Prérequis : IRM ou échographie < 3 mois)*	D

☐ **Autre raison de consultation non standardisée au formulaire ou modification d'une priorité clinique *(justification OBLIGATOIRE dans la section suivante)* :**

Priorité clinique

Impression diagnostique et renseignements cliniques obligatoires

Si prérequis exigé(s) :

☐ **Disponible(s) dans DSQ**

☐ **Annexé(s) à la présente demande**

Besoins spéciaux :

Identification du médecin référent et du point de service

Nom du médecin référent		N° de permis	
Ind. rég. N° de téléphone	N° de poste	Ind. rég. N° de télécopieur	
Nom du point de service			

Estampe

Signature

Date (année, mois, jour)

Médecin de famille : ☐ **Idem au médecin référent** ☐ **Usager sans médecin de famille**

Nom du médecin de famille

Nom du point de service

Référence nominative (si requis)

Si vous désirez une référence à un médecin ou à un point de service en particulier

Légende

[1] Lien web pour liste des Guichets d'entrée régionaux pour les traumatismes crâniens cérébraux (TCC) légers : http://fecst.inesss.qc.ca/fileadmin/documents/Publications/Liste-contacts-reference-TCCL-decembre-2016.pdf

Alertes cliniques et raisons de consultation de priorité A ou B (liste non exhaustive)
**Communiquer avec le neurochirurgien de garde**
• Hémorragie intracrânienne<br>• Syndrome d'hypertension intracrânienne avec ou sans altération de l'état de conscience<br>• Apparition soudaine ou rapidement progressive d'un déficit neurologique (localisation cérébrale, médullaire ou queue de cheval ou radiculopathie aiguë avec déficit moteur significatif)<br>• Myélopathie aiguë ou subaiguë (compression médullaire) avec évolution rapide des symptômes<br>• Sténose carotidienne symptomatique<br>• Traumatisme cranio-cérébral (TCC)[1] ou spino-médullaire aigu modéré et sévère<br>• Fracture du crâne ou du rachis<br>• Tumeurs cérébrales intraparenchymateuses : métastases, gliomes ou autres<br>• Tumeurs du rachis intra-durales ou extra-durales (primaires ou métastatiques)
