# Supplementary material for: Appropriateness of specialized care referrals for LBP: a cross-sectional analysis
Source: Front Med (Lausanne). 2024 Jan 5;10:1292481. doi: 10.3389/fmed.2023.1292481 (PMC10797061; doi:10.3389/fmed.2023.1292481)
Supplement: Supplementary file 3 [file Data_Sheet_3.pdf]

Supplementary File 3 : Regression analysis

Variables	OR	95 % CI	p-value
**Social deprivation**			
1st quintile	1.67	0.53-2.98	0.58
2nd quintile	1.70	0.74-3.89	0.21
3rd quintile	2.75	1.05-7.18	0.04
4th quintile	1.58	0.61-4.11	0.35
5th quintile	1.05	0.46-2.37	0.91
**Motor deficits**			
Absence of motor deficits	282 (56.4)	191 (38.2)	<0.001

CI : Confidence interval; OR :Odds ratios
